# Trajectory of Depression among Prostate Cancer Patients: A Secondary Analysis of a Randomized Controlled Trial

**DOI:** 10.3390/cancers15072124

**Published:** 2023-04-02

**Authors:** Sumedha Chhatre, Joseph J. Gallo, Thomas Guzzo, Knashawn H. Morales, Diane K. Newman, Neha Vapiwala, Keith Van Arsdalen, Alan J. Wein, Stanley Bruce Malkowicz, Ravishankar Jayadevappa

**Affiliations:** 1 Department of Psychiatry, Perelman School of Medicine, University of Pennsylvania, Philadelphia, PA 19104, USA; 2 Corporal Michael J. Crescenz VAMC, Philadelphia, PA 19104, USA; 3 Leonard Davis Institute of Health Economics, University of Pennsylvania, Philadelphia, PA 19104, USA; 4 Department of Mental Health, Bloomberg School of Public Health, Johns Hopkins University, Baltimore, MD 21205, USA; 5 Urology Division, Department of Surgery, Perelman School of Medicine, University of Pennsylvania, Philadelphia, PA 19104, USA; 6 Department of Biostatistics, Epidemiology and Informatics, Perelman School of Medicine, University of Pennsylvania, Philadelphia, PA 19104, USA; 7 Department of Radiation Oncology, Perelman School of Medicine, University of Pennsylvania, Philadelphia, PA 19104, USA; 8 Department of Medicine, Perelman School of Medicine, University of Pennsylvania, Philadelphia, PA 19104, USA; 9 Abramson Cancer Center, University of Pennsylvania, Philadelphia, PA 19104, USA

**Keywords:** localized prostate cancer, prostate cancer risk groups, depression, decision regret, longitudinal assessment

## Abstract

**Simple Summary:**

Prostate cancer is the most common malignancy (after skin cancer) in men, and also the most common cancer among cancer survivors in the US. We assessed the long-term burden of depression across three prostate cancer risk groups, and evaluated the association between regret and long-term depression. A large proportion of localized prostate cancer patients continued to experience long-term depression. The proportion with high depression increased over time for all risk groups. Higher regret at 24-month follow-up was significantly associated with high depression at 24-month follow-up, after adjusting for covariates. Patient-centered survivorship care strategies are needed to address depression and regret, and improve outcomes in prostate cancer care.

**Abstract:**

**Background:** While psychological difficulties, such as depression, among prostate cancer patients are known, their longitudinal burden remains understudied. We assessed the burden of depression across low-, intermediate- and high-risk prostate cancer groups, and the association between regret and long-term depression. **Methods:** Secondary analysis of data from a multi-centered randomized controlled study among localized prostate cancer patients was carried out. Assessments were performed at baseline, and at 3-, 6-, 12- and 24-month follow-up. Depression was assessed using the Center for Epidemiologic Studies Depression (CES-D) scale. A CES-D score ≥ 16 indicates high depression. Regret was measured using the regret scale of the Memorial Anxiety Scale for Prostate Cancer (MAX-PC). The proportion of patients with high depression was compared over time, for each risk category. Logistic regression was used to assess the association between regret, and long-term depression after adjusting for age, race, insurance, smoking status, marital status, income, education, employment, treatment, number of people in the household and study site. **Results:** The study had 743 localized prostate cancer patients. Median depression scores at 6, 12 and 24 months were significantly larger than the baseline median score, overall and for the three prostate cancer risk groups. The proportion of participants with high depression increased over time for all risk groups. Higher regret at 24-month follow-up was significantly associated with high depression at 24-month follow-up, after adjusting for covariates. **Conclusions:** A substantial proportion of localized prostate cancer patients continued to experience long-term depression. Patient-centered survivorship care strategies can help reduce depression and regret, and improve outcomes in prostate cancer care.

## 1. Introduction

Prostate cancer is the most common type of cancer (after skin cancer) in men in the United States. In 2023, there will be 288,300 new cases of prostate cancer and 34,700 prostate cancer-related death [1]. Currently, there are approximately 3,253,416 prostate cancer survivors in the United States [1]. Cancer diagnosis is a source of great distress, anxiety and depression for patients [2,3,4]. Estimates of the rate of depression accompanying prostate cancer diagnosis vary. In an analysis of Surveillance, Epidemiology and End Results (SEER) Medicare-linked data, 8.54% of newly diagnosed prostate cancer patients had a depression diagnosis [5]. This estimate is likely to be conservative, because Medicare claims data have been found to have poor sensitivity for clinical diagnosis of depression [6]. A meta-analysis showed that the prevalence of depression among prostate cancer patients ranged from 14 to 18% [7]. Studies have uniformly demonstrated the association between depression in prostate cancer and increase in the use of health services, poor quality of life and higher mortality [5,8,9,10,11,12]. One study showed that prostate cancer patients with depression who received androgen deprivation therapy experienced elevated periods of hospitalization [13]. Depression in patients with prostate cancer may be an unidentified factor in the variability in care, contributing to poorer long-term treatment outcomes [7].

Previous studies that examined the levels of depression among prostate cancer patients have been limited by small sample sizes, homogenous sample, reliance on claims data for depression diagnosis, or a cross-sectional design [7,8]. For example, few studies have employed longitudinal data after treatment [11,12]. Initial symptoms may change over time, so cross-sectional associations provide only a partial picture. Pre-treatment symptoms and traits are important to consider, since symptoms of depression may be related to what treatment option the patient selects [14,15]. Treatment decision regret may also contribute to the persisting longitudinal presence of depression [16]. Decision regret is the undesirable emotion of distress following a decision, and can manifest when the outcome of a decision is compared with the likely outcome of an unchosen alternative [17,18]. Thus, when a treatment decision is made in an uncertain or preference-sensitive situation, such as prostate cancer, it may lead to decision regret [9,17,19,20,21,22,23,24].

While studies have examined the longitudinal psychosocial consequences of curative treatments in men with prostate cancer [25], few have assessed the association between decision regret and depression among prostate cancer patients. In this paper, we present a secondary analysis using data from a randomized controlled trial of decision-making strategies for men with localized prostate cancer. Our objective is two-fold. First, we examine longitudinally, the proportion of those with high depression across three prostate cancer risk groups. Second, we assess the association between long-term depression and decision regret in our cohort of prostate cancer patients, for three different prostate cancer risk groups.

## 2. Materials and Methods

### 2.1. Study Design and Conduct

The overall study methodology has been described previously [26,27,28,29]. Briefly, in this multi-centered randomized controlled trial study, the intervention was a web-based, Patient Preferences for Prostate Cancer Care (PreProCare) instrument, that uses an adaptive choice based a conjoint (ACBC) analysis technique for preference assessment. The result of the PreProCare intervention was a list of five attributes that the participant valued most. All participants completed self-administered outcome assessments at baseline (prior to the intervention) and at the 3-, 6-, 12- and 24-month follow-up. Local institutional review boards approved the study. 

### 2.2. Study Participants

#### 2.2.1. Study Sites

The University of Pennsylvania (UPenn) was the primary and coordinating site (site 1). Site 2 was the Corporal Michael J. Crescenz Veterans Administration Medical Center (CMCVAMC) and Site 3 was Fox Chase Cancer Center/Temple University Hospital (FCCC). All study sites were located in Philadelphia, PA, USA. Based on sample size estimates, the total target accrual goal was 720 participants. The power calculations for specific aims assumed the availability of 720 participants who were eligible for randomization into one of the two intervention groups. We assumed a conservative intra-class correlation of 0.3, 4 follow-up measures per subject, and a two-sided level of significance of α = 0.05. The sample size was adjusted to accommodate a 10% missing or dropout rate by 24 months. We had 80% power to detect a 1.2-point difference in the prostate cancer index or SF-36 sub-scale [26].

#### 2.2.2. Study Eligibility Criteria

The study eligibility criteria were: (1) newly diagnosed with localized prostate cancer (low risk: PSA ≤ 10 ng/mL, Gleason ≤ 6, and stage T1c–T2a; intermediate risk: PSA > 10–≤ 20 ng/mL, or Gleason 7, or stage T2b; and high risk: PSA > 20 ng/mL, or Gleason score 8–10, or stage T2c); (2) treatment naïve (as of study entry); (3) age ≥ 18 years; and (4) able to provide informed consent. The exclusion criteria were: (1) distant, metastatic or un-staged prostate cancer at diagnosis; (2) unable to communicate in English; and (3) treatment for prostate cancer already underway. 

### 2.3. Recruitment and Randomization

Recruitment involved following steps: (1) obtaining consent from the patient’s urologist/physician for reviewing medical records; (2) determining eligibility via medical records; (3) screening to assess willingness to participate; and (4) obtaining informed consent and Health Insurance Portability and Accountability Act (HIPAA) permissions. The study biostatistician created randomization sequences for each site using a pseudo-random number generator with random blocking, varying in size from two to six. The treatment assignments were placed in sealed, opaque envelopes. Research coordinators opened the envelope and notified the participants of the group assignment. The study investigators were blinded to the group assignment. 

### 2.4. The PreProCare Intervention 

Participants in the intervention group completed the web-based, ACBC instrument, PreProCare, to assess their individual preferences. Briefly, this three-part tool was arranged as follows: a brief introduction to the instrument was provided in part one. In the second part, the participants ranked the attributes of various treatments (‘not important’ to ‘extremely important’). In the third part, choice scenarios, consisting of combinations of attributes, were presented based on the attribute ranking, and participants selected the combination that they most preferred. At end of the task, a graph and a list of the five attributes most preferred by the participant were generated. The participant had the option to have a printout of the output to share with his provider. On average, the PreProCare instrument required about 30 min to complete. Usual care group participants received standard care. 

### 2.5. Assessments

*Depression:* Depression was measured at baseline and all follow-up time points using the Center for Epidemiologic Studies Depression (CES-D) scale. The CES-D is a 20-item, self-report scale used to identify depression in the general population. It covers major components of depression, with an emphasis on affective components: depressed mood; feelings of guilt and worthlessness; feelings of helplessness and hopelessness; psychomotor retardation; loss of appetite; and sleep disorders [30]. The score ranges from 0 to 60, and a higher score indicates higher depression level. A score of 16 or more is indicative of ‘high’ depression.

*Regret:* Regret was measured using the five-item regret subscale of the Memorial Anxiety Scale for Prostate Cancer (MAX-PC) [31,32,33]. The score ranges from 0 to 100, with a higher score indicating greater decisional regret. 

*Treatment:* Data on primary treatment, such as active surveillance, open radical prostatectomy, robot-assisted radical prostatectomy and radiation therapy (intensity modulated radiation therapy, brachytherapy, or proton therapy) were obtained via medical charts and a self-report.

*Other covariates:* Self-reported data on age, income, race and ethnicity, education, marital status and employment were obtained at baseline. Data on height, weight, smoking status and health insurance were abstracted from electronic medical records. Data on Prostate Specific Antigen (PSA) levels, TNM stage, grade and histology, and the Gleason score were also abstracted from electronic medical records. 

### 2.6. Statistical Analysis

First, we compared the socio-demographic and clinical variables between the three prostate cancer risk groups. We then longitudinally examined the proportion of patients with ‘high depression’ for the three prostate cancer risk groups. Finally, we conducted logistic regression to assess the association between high depression and decision regret at 24-month follow-up, after adjusting for covariates. The covariates were operationalized as follows: regret score, age and number of people in the household were continuous variables; treatment was surgery, radiation, or active surveillance (reference category); race was white, African American, or other (reference category); education was college and higher, or some college (reference category); marital status was married or other (reference category); employment status was employed (full/part time), or other (reference category); income was ≤USD 40,000, USD 40–75,000, or >USD 75,000 (reference category); smoking was never, or current/ever (reference category); health insurance was Medicare/Medicaid, or other (reference category); and the study site was UPenn or FCCC/CMCVAMC (reference category). We conducted separate analysis for each prostate cancer risk group.

## 3. Results

We recruited 743 localized prostate cancer patients for the study. Comparison of baseline characteristics across the three prostate cancer risk groups is presented in Table 1. Age, number of people in the household, race and ethnicity, insurance, smoking status, marital status, income, education and employment were comparable between the three prostate cancer risk groups. The distribution of treatment varied across the prostate cancer risk groups (*p* < 0.0001). The low-risk group had the highest proportion of those on active surveillance, compared to intermediate- and high-risk groups (53.9% vs. 18.2% and 11.6%, respectively). On the other hand, the high-risk group had the highest proportion of those treated with surgery, compared to the low- and intermediate-risk groups (69.8% vs. 29.1% and 57.9%, respectively). 

The distribution of CES-D scores over time and by prostate cancer risk group is presented in Table 2. We used paired Wilcoxon signed rank test of the medians to test the difference in the medians (baseline and 3 months, baseline and 6 months, baseline and 12 months and baseline and 24 months). The results indicated that the medians at 6, 12 and 24 months were significantly different from baseline median values, overall and for the three prostate cancer risk groups. The mean regret score at 3 month was 9.4 (standard deviation (SD) 17.3), 8.1 (SD 16.6) at 6 month, 7.2 (SD 15.2) at 12 month and 7.7 (SD 15.7) at 24 month.

**Figure 1:**
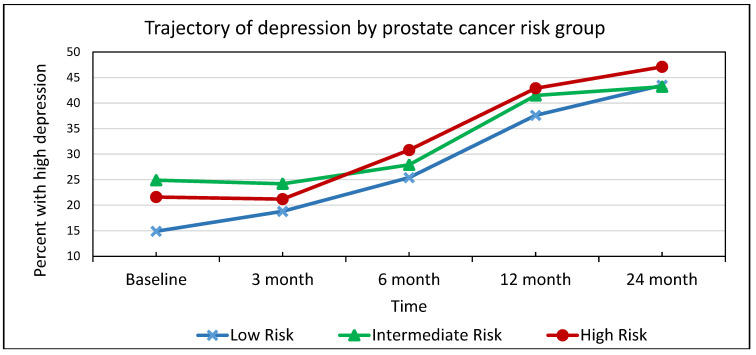
Trajectory of high depression by prostate cancer risk group *. * The increase in the proportion of patients with high depression over time was statistically significant for all three prostate cancer risk groups (0.05 level).

As seen in Figure 1, the proportion of patients with high depression increased between baseline and 24 months for risk groups. For the low-risk group, the proportion with high depression was 14.9% baseline and 43.5% at 24-month. These proportions were 24.9% and 43.2%, respectively, for the intermediate-risk group; and 21.6% and 47.1%, respectively, for the high-risk group. The increase in the proportion of patients with high depression over time was statistically significant for all three prostate cancer risk groups (0.05 level.)

In Table 3, we present the results of the multivariable logistic regression for association between 24-month depression and regret, by prostate cancer risk group. We observed that for all prostate cancer risk groups, regret was significantly associated with the risk of high depression. For one unit increase in the 24-month regret score, there was a 4 percent increase in the risk of high depression at 24-month follow-up, on average, after adjusting for age, race, insurance status, smoking status, marital status, income, education, employment, treatment, study site and the number of people in the household.

## 4. Discussion

We observed that among newly diagnosed prostate cancer patients undergoing treatment or those on active surveillance: (1) median depression scores at 6, 12 and 24 months were significantly higher than the baseline median score, overall and for the three prostate cancer risk groups; (2) the proportion with high depression increased over time (between baseline and 24-month follow-up), for all prostate cancer risk groups; and (3) higher regret was significantly associated with high depression at the 24-month follow-up, after adjusting for age, race, insurance status, smoking status, marital status, income, education, employment, treatment, study site and the number of people in the household.

Among patients living with other types of cancers, such as breast cancer [34,35,36,37], head and neck cancer [38,39,40], and esophageal cancer [41,42], depression and anxiety were found to be the most common problems, and were associated with impaired outcomes [40,43,44,45]. The research focusing on the association between depression and regret among cancer patients is limited. Among breast cancer patients undergoing reconstructive procedures, increased depression and lower satisfaction with information were associated with an increased likelihood of experiencing regret [46]. One study of adolescent and young adult cancer patients showed an association between regret and negative psychological outcomes, including anxiety and depression [47].

Our results make an important contribution to the limited existing literature on the assessment of the association between depression and treatment decision regret. As most men treated for localized prostate cancer can expect to live many years after diagnosis, the treatment-related morbidities are often experienced over an extended period of time, leading to treatment regret that can have long-term negative consequences on patients’ quality of life and depression [21,48]. Androgen deprivation therapy was shown to be associated with an increased risk of diagnosis of depression [49]. With increased survivorship and burden of disease, it is important to consider the physical and mental health of prostate cancer survivors. Previous studies found that the rates of depressive symptoms among prostate cancer survivors ranged from 16 to 30% [5,7,8,25,50].

Research has demonstrated that a large proportion of patients with prostate cancer experience some regret after treatment, and this regret tends to increase over time [21,22,23,24,51,52]. Diefenbach and Mohamed (2009) found that prostate cancer survivors who were regretful about their treatment choice(s) had a lower quality of life compared to those who were not regretful [53]. Previous studies have also found that men who had a passive role in treatment decision-making had higher treatment regret than those who assumed an active or collaborative role, and that among those who participated in medical decision-making, 94% did not experience treatment regret [54]. Our results are in concordance with research that reported an association between treatment decision regret and depression [24,25]. While the evidence for the impact of treatment regret on depression among prostate cancer survivors is evolving, its contribution can be important. Davison and Goldenberg (2003) found that emotional functioning was significantly better at follow-up among prostate cancer patients who participated in their treatment decision than those who did not [55]. In a longitudinal study of patients undergoing prostatectomy, shared decision-making was protective against regret, and patients with higher depression were more likely to have regret [24]. This suggests that treatment regret can have implications for depression.

We would like to note the strength and limitations of our study. The strength of our study is that this is a secondary analysis of one of the largest multicenter randomized controlled study that assessed self-reported regret and depression, using valid instruments over a 24-month follow-up period. Some of the limitations of our study are as follows: our study sites were urban academic institutions. Between baseline and 24 months, there were missing data due to lost to follow-up, as well as due to missing data. Most of the participants were married, college graduates and almost two-thirds had an annual household income of USD 75,000 or higher. Our results showed that among low-risk group participants, smoking status and income was associated with depression. Thus, future studies in patients with diverse characteristics and in different clinical settings are needed to enhance the generalizability of our findings.

## 5. Conclusions

Our study has several important clinical implications. In this secondary analysis of a large randomized controlled study, we assessed the burden of depression, and the association between decision regret and depression in localized prostate cancer patients. We observed that a substantial proportion of localized prostate cancer patients continued to experience long-term depression, irrespective of their prostate cancer risk group status. In addition, a positive association between decision regret and depression was noted, albeit the size of the effect was small. Studies, including our prior research, have demonstrated that depression in prostate cancer patients is associated with impaired outcomes of care. Thus, effective survivorship care strategies for prostate cancer must address depression and regret. Patient-centered strategies that facilitate patient participation in decision-making can help lower decision regret. Screening and surveillance for depression must be incorporated into clinical care of prostate cancer. Similarly, coordinated strategies are necessary for improving depression treatment uptake and retention in depression care.

## Figures and Tables

**Table 1:** Demographics and clinical variables across prostate cancer risk groups.

		Low Risk (n = 254)	Intermediate Risk (n = 247)	High Risk (n = 225)	*p*-Value
Mean age, years (SD)		63.9 (8.4)	63.6 (7.4)	63.6 (7.4)	0.8193
Mean Number of people in the household (SD)		2.3 (0.98)	2.2 (0.96)	2.5 (1.12)	0.0698
Race/Ethnicity	White	198 (79.8)	194 (80.8)	188 (87.0)	0.3005
	African American	40 (16.1)	36 (15.0)	23 (10.7)	
	Other	10 (4.0)	10 (4.2)	5 (2.3)	
Insurance	Medicare/Medicaid	106 (42.9)	83 (33.5)	84 (38.8)	0.1326
	Managed care	109 (44.1)	138 (56.3)	109 (50.5)	
	Private	32 (12.9)	24 (9.8)	23 (10.5)	
Smoking status	No	147 (58.1)	145 (58.7)	129 (58.4)	0.4850
	Yes	18 (7.1)	13 (5.3)	21 (9.5)	
	Prior history	88 (34.8)	89 (36.0)	71 (32.1)	
Marital status	Married	203 (82.2)	186 (77.2)	186 (84.9)	0.0942
	Other	44 (17.8)	55 (22.8)	33 (15.1)	
Income	≤USD 40,000	41 (19.8)	30 (14.7)	28 (14.4)	0.4627
	USD 40,000–75,000	46 (22.2)	41 (20.1)	41 (21.0)	
	>USD 75,000	120 (57.9)	133 (65.2)	126 (64.6)	
Education	Some college	84 (39.3)	81 (38.6)	80 (39.4)	0.9828
	College and other	130 (60.8)	129 (61.4)	123 (60.6)	
Employment	Full/part time	113 (52.8)	126 (60.3)	123 (60.3)	0.1981
	Other	101 (47.2)	83 (39.7)	81 (39.7)	
Treatment Type	Active surveillance	137 (53.9)	45 (18.2)	26 (11.6)	<0.0001
	Surgery	74 (29.1)	143 (57.9)	157 (69.8)	
	Radiation	19 (7.5)	41 (16.6)	32 (14.2)	
Site	UPenn	174 (68.5)	195 (78.9)	173 (77.6)	<0.0001
	VA	18 (7.1)	1 (0.4)	3 (1.3)	
	FCCC	62 (24.4)	51 (20.6)	49 (21.1)	

Note. Data are presented as a no. (%), unless otherwise indicated. Missing values: prostate cancer risk group (n = 17), age (n = 19), number of people in household (n = 98), race/ethnicity (n = 22), insurance (n = 18), smoking (n = 5), marital status (n = 19), income (n = 120), education (n = 99), employment (n = 99) and treatment type (n = 52). Abbreviations: SD, standard deviation; UPenn, University of Pennsylvania; CMCVAMC, Corporal Michael J. Crescenz Veterans Administration Medical Center; FCCC, Fox Chase Cancer Center.

**Table 2:** Distribution of CES-D scores over time, and by prostate cancer risk group.

	Baseline	3 m	6 m	12 m	24 m
ALL					
Mean	9.04	9.52	11.69	15.40	16.02
SD	8.12	8.21	7.99	5.55	4.91
Median	7.00	7.00	12.00	14.00	15.00
Wilcoxon signed rank test of medians—compared to baseline median value		*p*-value 0.4219	*p*-value < 0.0001	*p*-value < 0.0001	*p*-value < 0.0001
Low Risk					
Mean	8.12	9.20	11.42	15.15	16.02
SD	7.73	8.67	7.57	4.82	4.79
Median	6.00	6.00	12.00	14.00	14.00
Wilcoxon signed rank test of medians—compared to baseline median value		*p*-value 0.0811	*p*-value < 0.0001	*p*-value < 0.0001	*p*-value < 0.0001
Intermediate Risk					
Mean	9.60	10.37	11.93	15.87	16.06
SD	8.48	8.69	8.55	5.66	5.09
Median	7.00	9.00	13.00	14.00	15.00
Wilcoxon signed rank test of medians—compared to baseline median value		*p*-value 0.8132	*p*-value 0.0049	*p*-value < 0.0001	*p*-value < 0.0001
High Risk					
Mean	9.30	9.09	11.59	15.15	15.96
SD	7.94	7.27	7.94	6.14	4.87
Median	7.00	7.00	12.00	15.00	15.00
Wilcoxon signed rank test of medians—compared to baseline median value		*p*-value 0.8691	*p*-value < 0.0001	*p*-value < 0.0001	*p*-value < 0.0001

Abbreviations: SD, standard deviation.

**Table 3:** Multivariable logistic regression for association between 24-month depression and regret, by prostate cancer risk group.

	Dependent Variable: High Depression at 24 Months								
	Low Risk			Intermediate Risk			High Risk		
	OR	95% CI	*p*-Value	OR	95% CI	*p*-Value	OR	95% CI	*p*-Value
Regret score	1.06	1.01, 1.11	0.0111	1.04	1.01, 1.08	0.0188	1.04	1.01, 1.07	0.0209
Age	0.98	0.89, 1.06	0.5402	1.03	0.96, 1.10	0.4154	0.96	0.89, 1.04	0.3199
Number of people in household	1.44	0.86, 2.42	0.1647	1.02	0.64, 1.65	0.9116	1.17	0.75, 1.83	0.4780
Smoking									
Never	0.22	0.10, 0.59	0.0031	0.51	0.22, 1.17	0.1136	1.06	0.48, 2.23	0.8900
Current/ever (ref)	-	-	-	-	-	-	-	-	-
Insurance									
Medicare/Medicaid	0.37	0.12, 1.17	0.0903	0.93	0.36, 2.37	0.8723	1.74	0.69, 4.39	0.2385
Other (ref)	-	-	-	-	-	-	-	-	-
Treatment									
Surgery	0.71	0.25, 2.05	0.5303	0.67	0.24, 1.87	0.4492	1.03	0.30, 3.61	0.9613
Radiation	0.39	0.07, 2.26	0.2956	0.44	0.12, 1.55	0.1998	2.47	0.53, 11.58	0.2497
Active surveillance (ref)	-	-	-	-	-	-	-	-	-
Race									
White	1.82	0.10, 35.70	0.6922	1.61	0.21, 12.24	0.6480	1.52	0.07, 32.60	0.7882
African American	2.37	0.07, 78.90	0.6304	3.34	0.29, 38.30	0.3322	3.26	0.12, 91.60	0.4875
Other (reference)	-	-	-	-	-	-	-	-	-
Education									
College and more	0.88	0.29, 2.59	0.8105	1.74	0.69, 4.36	0.2408	1.83	0.72, 4.65	0.2023
Some college (ref)	-	-	-	-	-	-	-	-	-
Marital status									
Married	0.45	0.09, 2.23	0.3305	0.67	0.21, 2.11	0.4957	0.97	0.24, 3.94	0.9678
Other (ref)	-	-	-	-	-	-	-	-	-
Employment status									
Employed	1.67	0.49, 5.69	0.4154	0.75	0.31, 1.85	0.5363	1.19	0.49, 2.92	0.6943
Other (ref)	-	-	-	-	-	-	-	-	-
Income									
≤USD 40,000	1.97	0.37, 10.55	0.4287	3.80	0.85, 17.72	0.4249	0.86	0.19, 3.76	0.8419
USD 40–75,000	4.39	1.29, 14.98	0.0179	0.65	0.22, 1.88	0.0803	2.74	0.96, 7.75	0.0579
>USD 75,000 (ref)	-	-	-	-	-	-	-	-	-
Study Site									
UPenn	1.78	0.59, 5.34	0.2975	0.55	0.22, 1.36	0.1930	1.06	0.48, 2.34	0.5935
FCCC/VA (ref)	-	-	-	-	-	-	-	-	-

Abbreviations: UPenn, University of Pennsylvania; CMCVAMC, Corporal Michael J. Crescenz Veterans Administration Medical Center; FCCC, Fox Chase Cancer Center. OR: odds ratio. CI: confidence interval.

## Data Availability

The data were collected as part of a large randomized controlled study and contain sensitive information. As such, the data cannot be shared.
